# The influence of hippocampal atrophy on the cognitive phenotype of dementia with Lewy bodies

**DOI:** 10.1002/gps.4719

**Published:** 2017-04-20

**Authors:** Greg J. Elder, Karen Mactier, Sean J. Colloby, Rosie Watson, Andrew M. Blamire, John T. O'Brien, John‐Paul Taylor

**Affiliations:** ^1^ Institute of Neuroscience Newcastle University, Campus for Ageing and Vitality Newcastle upon Tyne UK; ^2^ The Florey Institute of Neuroscience and Mental Health Parkville VIC Australia; ^3^ Newcastle Magnetic Resonance Centre Newcastle University Newcastle upon Tyne UK; ^4^ Department of Psychiatry University of Cambridge Cambridge UK

**Keywords:** hippocampus, dementia with Lewy bodies, Alzheimer's disease, magnetic resonance imaging

## Abstract

**Objective:**

The level of hippocampal atrophy in dementia with Lewy bodies (DLB) is typically less than that observed in Alzheimer's disease (AD). However, it is not known how the cognitive phenotype of DLB is influenced by hippocampal atrophy or the atrophy of adjacent medial temporal lobe structures.

**Methods:**

Dementia with Lewy bodies (*n* = 65), AD (*n* = 76) and control (*n* = 63) participants underwent 3T magnetic resonance imaging and cognitive Cambridge Cognitive Examination and Mini‐Mental State Examination (CAMCOG and MMSE) assessments. Hippocampal volume, and parahippocampal, entorhinal and temporal pole cortical thickness, was compared between groups. Regression models were used to investigate whether hippocampal volume and cortical thickness associated with global cognition and cognitive subdomains.

**Results:**

Dementia with Lewy bodies, AD and control participants showed significantly different hippocampal, parahippocampal and entorhinal cortical thinning, where atrophy was greatest in AD and intermediate in DLB. Temporal pole thickness was reduced in DLB and AD compared with control participants. In DLB, but not AD, hippocampal volume associated with total CAMCOG, CAMCOG memory and MMSE scores. In DLB, parahippocampal, entorhinal and temporal pole thickness associated with total CAMCOG and CAMCOG memory scores, parahippocampal thickness associated with MMSE scores, and entorhinal thickness associated with CAMCOG executive function scores.

**Conclusions:**

In this large sample, these results are in agreement with other studies indicating that hippocampal atrophy is less severe in DLB than AD. Hippocampal atrophy and medial temporal lobe cortical thickness were associated with the severity of cognitive symptoms, suggesting that atrophy in these structures, as a potential proxy of AD pathology, may partly mediate specific DLB cognitive symptoms. © 2017 The Authors. *International Journal of Geriatric Psychiatry* Published by John Wiley & Sons Ltd.

## Introduction

Dementia with Lewy bodies (DLB) is the second most common cause of dementia in older people after Alzheimer's disease (AD), and accounts for 15–20% of all dementia cases at post mortem (McKeith *et al.,* 2005). DLB is characterised by symptoms including motor and neuropsychiatric problems, executive dysfunction and visuospatial deficits, visual hallucinations, autonomic dysfunction and marked fluctuations in alertness (McKeith *et al.,* 2005; McKeith, 2006). There are differences between the symptom profile of DLB and AD, where memory problems are typically more pronounced in AD, and attentional and visuoperceptual difficulties are more prominent in DLB (Calderon *et al.,* 2001; Ferman *et al.,* 2006).

To date, the use of magnetic resonance (MR) imaging has been useful in understanding the differences between DLB and AD, despite the low‐to‐intermediate co‐existence of AD neuropathology in individuals with DLB (Merdes *et al.,* 2003; McKeith *et al.,* 2005; Schneider *et al.,* 2012). One particular region of relevance is the hippocampus, which is located within the medial temporal lobe and has a functional role in declarative memory, emotional memory and spatial navigation (Eichenbaum, 2004; Strange *et al.,* 2014). Hippocampal atrophy is a marker of AD pathology, as hippocampal volume has been shown to negatively associate with the tau and amyloid beta burden in AD (Apostolova *et al.,* 2015) and hippocampal atrophy can predate a diagnosis of dementia by a period of up to several years (Tondelli *et al.,* 2012; de Flores *et al.,* 2015) also making it a potentially important prodromal biomarker.

Hippocampal AD pathology is also a feature of DLB, as previous imaging studies have consistently demonstrated that the level of hippocampal atrophy observed in DLB is less than in AD (Hashimoto *et al.,* 1998; Tam *et al.,* 2005; Burton *et al.,* 2009; Watson *et al.,* 2012), and the level of atrophy relates to concurrent AD pathology measured post mortem (Burton *et al.,* 2009; Kantarci *et al.,* 2012). The extent of the atrophy may also influence the subsequent clinical course of DLB, as individuals with higher levels of hippocampal atrophy have a shorter survival time compared with those with lower levels of atrophy (Graff‐Radford *et al.,* 2016).

It is not known how hippocampal atrophy, or the thickness of adjacent medial temporal lobe structures, influences the cognitive phenotype of DLB. Three relevant subregions are (i) the parahippocampal gyrus, which has a predominant role in episodic memory and an involvement in visuospatial processing (Aminoff *et al.,* 2013); (ii) the entorhinal cortex, which relays information from sensory cortical areas of the hippocampus, and from the hippocampus to neocortical storage areas (Heinemann *et al.,* 2000); and (iii) the temporal pole, which has a role in cognition and memory function and particularly declarative memory (Blaizot *et al.,* 2010). Whilst one previous study showed that compared with healthy control participants, DLB participants showed reductions in the thickness of the right entorhinal, right perirhinal and right parahippocampal areas; there were no differences compared with AD participants and no associations between these regions and cognitive variables in DLB (Delli Pizzi *et al.,* 2016). However, the main limitation of this study was in the relatively modest sample size.

Therefore, the main aim of the present study was to investigate whether hippocampal atrophy, as a proxy of AD pathology, is associated with cognitive deficits in DLB, in a large sample of dementia patients. A secondary aim was to examine the association between the thickness of the parahippocampal gyrus, entorhinal cortex and temporal pole, and measures of cognition in DLB. It was hypothesised that DLB patients would display a level of hippocampal atrophy that was greater in severity than healthy control individuals but less than that observed in AD, and individuals with DLB would display a greater level of surface atrophy in adjacent medial temporal lobe subregions, as compared with healthy controls, but less than that observed in AD.

## Method

### Participants

A total of 204 participants (*M*_age_ = 77.75 years; SD_age_ = 6.76 years), including 76 with probable AD (McKhann *et al.,* 1984), 65 with probable DLB (McKeith *et al.,* 2005) and 63 similarly older healthy controls, were recruited from a community‐dwelling population of patients referred to Old Age Psychiatry, Geriatric Medicine or Neurology services. Control participants were recruited from the relatives and friends of patients with dementia and from a bank of volunteer participants held by Newcastle University. Exclusion criteria for all participants included contra‐indications for MR imaging, a previous history of alcohol or substance misuse, a significant neurological history or psychiatric illness, focal brain lesions, or the presence of other relevant severe or uncontrolled illnesses. Participants, or their nearest relative or carer, provided informed consent. The diagnosis of DLB was confirmed by two senior experienced clinicians. The study was approved by the local research ethics committee.

### Measures

Global cognition was assessed using the Mini Mental State Examination (MMSE; Folstein *et al.,* 1975) and the Cambridge Cognitive Examination (CAMCOG; Roth *et al.,* 1986). In DLB and AD patients, the severity of parkinsonian motor symptoms were assessed using Part III of the Unified Parkinson's Disease Rating Scale (UPDRS‐III; Goetz *et al.,* 2008), and with the help of a carer/informant, the severity of visual hallucinations and depressive symptoms in the previous four weeks were assessed using the Neuropsychiatric Inventory (NPI; Cummings *et al.,* 1994), and the severity of cognitive fluctuations in the previous four weeks were assessed using the Clinician Assessment of Fluctuation Scale (Walker *et al.,* 2000).

### Magnetic resonance imaging data acquisition

All participants underwent MR imaging within 2 months of completing neuropsychiatric assessments. T1‐weighted whole brain scans were obtained using a 3T Intera Achieva scanner (Philips Medical Systems, Eindhoven, Netherlands) with an eight‐channel head coil. The imaging sequence protocol was as follows: 3D MPRAGE, sagittal acquisition, 1‐mm isotropic resolution, matrix size of 240 (anterior–posterior) × 216 (superior–inferior) × 180 (right–left); repetition time = 8.3 ms, echo time = 4.6 ms; SENSE factor = 2; flip angle = 8°. The acquired volume was angulated such that the axial slice orientation was standardised to align with the AC–PC line.

### Calculation of hippocampal and total grey matter volumes and cortical thickness

Estimates of hippocampal and total grey matter (GM) volumes as well as regional cortical thickness of specific medial temporal lobe structures (parahippocampal gyrus, entorhinal cortex and temporal pole) were obtained for each participant using freesurfer (v.5.1.0, http://surfer.nmr.mgh.harvard.edu/). The technical aspects of these methods have been described elsewhere (Dale *et al.,* 1999; Fischl *et al.,* 1999; Fischl and Dale, 2000). In brief, the processing stream involves intensity non‐uniformity correction, Talairach registration, the removal of non‐brain tissue (skull stripping), segmentation of white matter (WM) and subcortical GM structures (Fischl *et al.,* 2002), tessellation of the GM‐WM boundary then surface deformation following GM‐cerebrospinal fluid (CSF) intensity gradients to optimally place GM‐WM and GM‐CSF borders (Dale *et al.,* 1999; Fischl and Dale, 2000). Once cortical models were generated, surface inflation, transformation to a spherical atlas and parcellation of the cerebral cortex into regions based on gyral and sulcal structure were undertaken (Makris *et al.,* 2006). This technique used both intensity and continuity information from the entire 3D MR volume in the segmentation and deformation procedures to produce representations of cortical thickness, calculated as the closest distance from the GM‐WM to GM‐CSF boundaries at each vertex on the tessellated surface (Fischl *et al.,* 2004). Total GM volume was calculated as the sum of subcortical, cortical (left and right) and cerebellum GM.

Visual inspection of images at each step of the freesurfer processing stream was carefully conducted by a single operator (S. J. C.) to ensure segmentation accuracy of the subcortical structures, as well as WM/pial surfaces and tissue classifications. Images with segmentation errors, which were unable to be corrected, were excluded from subsequent analyses.

### Data analysis

Cambridge Cognitive Examination total, memory and executive function, MMSE, UPDRS‐III, NPI depression, NPI visual hallucinations and Clinician Assessment of Fluctuation Scale scores were compared between DLB, AD and control participants using a one‐way analysis of variance or independent‐samples *t*‐tests, where appropriate. Chi‐squared tests were used to assess group differences in age and gender. The volume of the left, right and total hippocampus (mm^3^) were compared between‐groups using an analysis of covariance, controlling for intracranial volume. Left, right and total cortical thickness measures of the parahippocampal gyrus, entorhinal cortex and temporal pole (mm) were compared between DLB, AD and control groups using a series of one‐way analysis of variances. *Post‐hoc* comparisons were Bonferroni‐corrected (*p* = .017), where appropriate.

Separate linear regression models were used, with the inclusion of age and intracranial volume as covariates, to investigate whether total hippocampal volume was associated with cognitive scores (CAMCOG total, CAMCOG memory, CAMCOG executive function and MMSE). In addition, linear regression models, with the inclusion of age as a covariate, examined whether total cortical thickness measures of the parahippocampal gyrus, entorhinal and temporal poles were associated with cognitive scores. Additional exploratory analyses were also conducted using linear regression models, with the inclusion of age as a covariate, to examine whether total parahippocampal, entorhinal and temporal pole cortical thickness was associated with other CAMCOG subscales (orientation, language, attention, calculation, praxis, abstract thinking and perception), and with three further memory subdomains (remote memory, recent memory and learning memory).

## Results

### Demographic and clinical results

Demographic and clinical results are shown in Table 1. DLB, AD and control groups did not differ in age or gender (*p* > .05). There were significant between‐group differences in CAMCOG total scores (*F*(2,203) = 132.18, *p* < .001, *η* ^2^_p_ = .57), CAMCOG memory (*F*(2,203) = 182.57, *p* < .001, *η* ^2^_p_ = .65) and CAMCOG executive function subscores (*F*(2,203) = 108.78, *p* < .001, *η* ^2^_p_ = .52). Compared with control participants, CAMCOG scores were significantly lower in DLB and AD, but there were no differences between the dementia groups. All three groups showed significant differences on CAMCOG memory scores, where control participants showed the lowest levels of impairment, DLB participants intermediate impairment and AD the greatest levels of impairment. For CAMCOG executive function scores, *post‐hoc* comparisons showed that DLB participants had lowest scores, AD participants displayed intermediate scores and controls had the highest scores. MMSE scores were significantly different between DLB, AD and control groups (*F*(2,203) = 101.97, *p* < .001, *η* ^2^_p_ = .50). *Post‐hoc* comparisons indicated that control participants obtained highest scores but that there were no differences between DLB and AD groups.

**Table 1:** Demographic and group characteristics

	DLB (*n* = 65)		AD (*n* = 76)		Control (*n* = 63)		Group comparisons
	Mean	SD	Mean	SD	Mean	SD	
Age	78.40	6.47	77.99	7.51	76.81	6.05	*p* > .05
Disease duration (months)	35.96	27.74	32.61	21.83	N/A	N/A	*p* > .05
Gender (male/female)	45/20		48/28		36/27		*p* > .05
CAMCOG total	67.94	15.27	67.09	12.96	96.76	3.77	*p* < .001^a^^,^ b
CAMCOG memory score	15.40	4.99	10.42	4.45	23.52	1.79	*p* < .001^a^^,^ b ^,^ c
CAMCOG executive function	11.66	5.15	13.67	4.64	22.36	2.95*	*p* < .001^a^^,^ b ^,^ d
MMSE	20.46	5.23	20.25	4.32	29.00	1.00*	*p* < .001^a^^,^ b
UPDRS‐III total	20.51	13.14	3.79	3.68	6.19	12.81*	*p* < .001
CAF	5.47	4.03	1.85	3.40	N/A	N/A*	*p* < .001
NPI hallucinations	2.30	2.55	.25	.81	N/A	N/A*	*p* < .001
NPI depression	1.17	2.42	.97	1.57	N/A	N/A	*p* > .05

AD, Alzheimer's disease; DLB, dementia with Lewy bodies; CAMCOG, Cambridge Cognitive Examination; CAF, Clinician Assessment of Fluctuation Scale; MMSE, Mini‐Mental State Examination; NPI, Neuropsychiatric Inventory; SD, standard deviation; UPDRS‐III, Unified Parkinson's Disease Rating Scale (Part III).

a DLB < control.

b AD < control.

c AD < DLB.

d DLB < AD.

* *p* < .001.

As expected, there were significant differences between all three groups in terms of UPDRS‐III scores (*F*(2,199) = 48.73, *p* < .001, *η* ^2^_p_ = .33), where motor impairments were greater in DLB compared with AD and control participants. Similarly, cognitive fluctuations were more severe in DLB than in AD participants (*t*(100.71) = 5.30, *p* < .001). DLB participants also had significantly more visual hallucinations in the previous month compared with AD participants (*t*(59.70) = 5.66, *p* < .001) but not depressive symptoms (*t*(111) = .55, *p* > .05). Neuropsychiatric symptoms were not associated with hippocampal volume (all *p*‐values > .05).

### Magnetic resonance imaging

There were significant differences in intracranial volume between DLB, AD and control groups (*F*(2,204) = 4.46, *p* < .05, *η* ^2^_p_ = .04), where DLB participants had a significantly greater volume than control but not AD participants. Significant differences were observed in the left hippocampus (*F*(2,203) = 67.12, *p* < .001, *η* ^2^_p_ = .40), right hippocampus (*F*(2,203) = 52.95, *p* < .001, *η* ^2^_p_ = .35) and in total hippocampal volumes (*F*(2,203) = 65.53, *p* < .001, *η* ^2^_p_ = .40). *Post‐hoc* comparisons indicated that hippocampal volume was lowest in individuals with AD, moderate in individuals with DLB and greatest in control participants (*p* < .001; Table 2).

**Table 2:** Comparison of hippocampal volume between DLB, AD and control groups

	DLB (*n* = 65)		AD (*n* = 76)		Control (*n* = 63)		Group comparisons
	Mean	SD	Mean	SD	Mean	SD	
Left Hippocampus volume (mm^3^)	3062.00	596.26	2536.38	626.28	3564.75	469.21	*p* < .001^a^^,^ b ^,^ c
Right Hippocampus volume (mm^3^)	3121.40	580.33	2598.74	626.26	3539.70	558.45	*p* < .001^a^^,^ b ^,^ c
Total Hippocampus volume (mm^3^)	6183.40	1134.45	5135.12	1199.19	7104.44	981.17	*p* < .001^a^^,^ b ^,^ c
Total Intracranial Volume (mm^3^)	1318687.47	261299.23	1288641.50	247635.81	1198843.29	192283.20	*p* < .05^a^

AD, Alzheimer's disease; DLB, dementia with Lewy bodies; SD, standard deviation.

a DLB < control.

b AD < control.

c AD < DLB.

* *p* < .05.

** *p* < .01.

*** *p* < .001.

Significant differences in cortical thickness were observed between DLB, AD and control groups in the parahippocampal gyrus, left (*F*(2,203) = 20.03, *p* < .001, *η* ^2^_p_ = .17), right (*F*(2,203) = 20.13, *p* < .001, *η* ^2^_p_ = .17) and total thickness (*F*(2,203) = 23.88, *p* < .001, *η* ^2^_p_ = .19); in entorhinal areas, left (*F*(2,203) = 42.96, *p* < .001, *η* ^2^_p_ = .30), right (*F*(2,203) = 28.22, *p* < .001, *η* ^2^_p_ = .22) and total thickness (*F*(2,203) = 40.30, *p* < .001, *η* ^2^_p_ = .29); and temporal pole, left (*F*(2,203) = 8.44, *p* < .001, *η* ^2^_p_ = .08), right (*F*(2,203) = 12.67, *p* < .001, *η* ^2^_p_ = .11) and total thickness (*F*(2,203) = 13.73, *p* < .001, *η* ^2^_p_ = .12). *Post‐hoc* comparisons indicated a consistent difference in profile (controls > DLB > AD) with the exception of the temporal pole, where DLB and AD participants both significantly differed from controls but not from each other (Table 3).

**Table 3:** Comparison of medial temporal subregions between DLB, AD and control groups

	DLB (*n* = 65)		AD (*n* = 76)		Control (*n* = 63)		Group comparisons
	Mean	SD	Mean	SD	Mean	SD	
Left parahippocampal thickness (mm)	2.30	.35	2.12	.28	2.46	.32	*p* < .001^a^^,^ b ^,^ c
Right parahippocampal thickness (mm)	2.28	.30	2.15	.31	2.47	.28	*p* < .001^a^^,^ b ^,^ c
Left entorhinal thickness (mm)	2.79	.42	2.41	.42	3.01	.32	*p* < .001^a^^,^ b ^,^ c
Right entorhinal thickness (mm)	2.79	.47	2.47	.51	3.06	.39	*p* < .001^a^^,^ b ^,^ c
Left temporal pole thickness (mm)	3.32	.38	3.23	.49	3.51	.33	*p* < .001^a^^,^ b
Right temporal pole thickness (mm)	3.28	.42	3.14	.56	3.55	.41	*p* < .001^a^^,^ b
Total parahippocampal thickness (mm)	4.58	.60	4.27	.55	4.93	.54	*p* < .001^a^^,^ b ^,^ c
Total entorhinal thickness (mm)	5.58	.82	4.88	.86	6.07	.66	*p* < .001^a^^,^ b ^,^ c
Total temporal pole thickness (mm)	6.60	.72	6.37	.94	7.06	.61	*p* < .001^a^^,^ b

a DLB < control;

b AD < control;

c AD < DLB.

* *p* < .05.

** *p* < .01.

*** *p* < .001.

In DLB participants, total hippocampal volume was significantly associated with total CAMCOG (*R* ^2^ = .21, *F*(3,64) = 5.24, *p* < .01), CAMCOG memory (*R* ^2^ = .38, *F*(3,64) = 12.25, *p* < .001), CAMCOG executive function (*R* ^2^ = .13, *F*(3,64) = 3.07, *p* < .05) and MMSE scores (*R* ^2^ = .13, *F*(3,64) = 2.95, *p* < .05; Table 4). In AD, total hippocampal volume was not associated with total CAMCOG, CAMCOG memory, CAMCOG executive function, or MMSE scores (all *p*‐values > .05; Table S1).

**Table 4:** Associations between total hippocampal volume and cognition in DLB (*n* = 65)

	*B*	*SE B*	*β*
CAMCOG total	.01	.00	.47**
CAMCOG memory	.00	.00	.68***
CAMCOG executive function	.00	.00	.36
MMSE	.00	.00	.37**

CAMCOG: Cambridge Cognitive Examination, MMSE: Mini‐Mental State Examination

* *p* < .05,

** *p* < .01,

*** *p* < .001.

In DLB, total parahippocampal thickness was associated with total CAMCOG (*R* ^2^ = .15, *F*(2,64) = 5.64, *p* < .01), CAMCOG memory (*R* ^2^ = .21, *F*(2,64) = 8.05, *p* < .01) and MMSE (*R* ^2^ = .12, *F*(2,64) = 4.30, *p* < .05), but not CAMCOG executive function scores (*p* > .05). Total entorhinal thickness in DLB was associated with total CAMCOG (*R* ^2^ = .22, *F*(2,64) = 8.77, *p* < .001), CAMCOG memory (*R* ^2^ = .41, *F*(2,64) = 21.36, *p* < .001), CAMCOG executive function (*R* ^2^ = .11, *F*(2,64) = 3.93, *p* < .05) but not MMSE scores (*p* > .05). Total temporal pole thickness in the DLB group was associated with total CAMCOG (*R* ^2^ = .16, *F*(2,64) = 5.71, *p* < .01), CAMCOG memory (*R* ^2^ = .26, *F*(2,64) = 10.79, *p* < .001), but not CAMCOG executive function or MMSE scores (*p*‐values > .05; Table 5). In AD, parahippocampal, entorhinal and temporal pole thickness was not associated with total CAMCOG, CAMCOG memory, CAMCOG executive function or MMSE scores (all *p*‐values > .05; Table S2).

**Table 5:** Associations between medial temporal subregion cortical thickness and cognition in DLB (*n* = 65)

	*B*	*SE B*	*β*
Total parahippocampal thickness	9.54	3.02	.38**
CAMCOG total	3.15	.96	.38**
CAMCOG memory	1.72	1.08	.20
CAMCOG executive function	3.09	1.05	.36**
MMSE			
Total entorhinal thickness	8.93	2.22	.48***
CAMCOG total	3.78	.63	.63***
CAMCOG memory	2.22	.80	.36**
CAMCOG executive function	2.04	.82	.32
MMSE			
Total temporal pole thickness	8.14	2.56	.39**
CAMCOG total	3.13	.78	.46***
CAMCOG memory	1.45	.91	.20
CAMCOG executive function	1.75	.93	.24
MMSE	9.54	3.02	.38**

CAMCOG, Cambridge Cognitive Examination; DLB, dementia with Lewy bodies; MMSE, Mini‐Mental State Examination.

* *p* < .05.

** *p* < .01.

*** *p* < .001.

Examination of CAMCOG subscales indicated that in DLB participants, total parahippocampal thickness was associated with language expression, attention, calculation and all three memory subdomains (remote, recent and learning memory); entorhinal thickness was associated with orientation, language expression, and all three memory domains, and temporal pole thickness was associated with language expression, calculation and the recent memory and learning memory subdomains (model *p*‐values < .05).

## Discussion

The results of the present study indicate that hippocampal atrophy, as a proxy of AD pathology, is present in DLB but is less severe than in AD. DLB, AD and control groups showed significant differences in left, right and total parahippocampal, entorhinal and temporal pole surface thickness, where the thickness was greatest in controls, intermediate in DLB and lowest in AD. One exception to this was in the left temporal pole, where the surface thickness was greatest in controls but did not differ between DLB and AD groups. These results also indicated that hippocampal atrophy was associated with the severity of specific cognitive deficits in DLB, as total hippocampal volume was associated with total CAMCOG and MMSE scores, as global measures of cognition and with CAMCOG memory subscores. In DLB, parahippocampal surface thickness was associated with total CAMCOG, CAMCOG memory and MMSE scores; entorhinal surface thickness was associated with CAMCOG total, CAMCOG memory and executive function scores and temporal pole surface thickness was associated with CAMCOG total and CAMCOG memory scores. In AD, parahippocampal, entorhinal and temporal pole thickness was not associated with measures of cognition.

These results are in agreement with previous studies, which have observed that the hippocampus is relatively preserved in DLB as compared with AD (Hashimoto *et al.,* 1998; Tam *et al.,* 2005; Burton *et al.,* 2009; Watson *et al.,* 2012). The present study also indicates that other regions of the medial temporal lobe display an intermediate level of atrophy in DLB, specifically in the parahippocampal, entorhinal and temporal pole areas. With the exception of the left temporal lobe area, DLB and AD groups showed differential patterns of surface atrophy. These results are partially in agreement with a previous study that demonstrated that compared with controls, right entorhinal and parahippocampal thickness was reduced (Delli Pizzi *et al.,* 2016). However, this previous study may have been underpowered and thus unable to detect subtle differences between DLB and AD. In the current study, no relationship was observed between surface thickness and cognitive measures in AD. Whilst relationships between cognition and medial temporal atrophy, and hippocampal volume, have been observed in previous studies in individuals with mild AD (Dickerson *et al.,* 2009; Arlt *et al.,* 2013), the lack of a relationship in the present study may reflect differences in AD participants, compared with the present sample, and in the differing neuropsychological measures used in these studies.

Overall, these results indicate that despite the relative preservation of the hippocampus in DLB, hippocampal atrophy is associated with global cognitive and memory impairment, and these relationships may represent AD pathology in the hippocampus. However, other pathologies may contribute to the cognitive symptoms of DLB and should be investigated further; one study has previously observed that cognitive decline in LBD may be a consequence of alpha‐synuclein, amyloid‐beta and phosphorylated tau pathology (Howlett *et al.,* 2015), and additionally, one study of the medial temporal lobe showed that alpha‐synuclein pathology, rather than AD pathology, had a role in the atrophy of the amygdala (Burton *et al.,* 2012). Hippocampal volume showed the strongest association with the CAMCOG memory score, which is in keeping with the memory function of the hippocampus (Eichenbaum, 2004; Strange *et al.,* 2014). These results also indicate that atrophy to adjacent medial temporal lobe subregions can also influence the DLB cognitive phenotype, with an overlap of symptoms: parahippocampal surface thickness was associated with CAMCOG cognition and memory subscores, but not executive function, the entorhinal surface thickness was associated with total CAMCOG scores, as well as CAMCOG memory and executive function subscores. No association was observed between the MMSE, as a measure of global cognition, and entorhinal thickness. Although AD pathology typically first appears in entorhinal regions (Schonheit *et al.,* 2004) and the entorhinal cortex projects to the hippocampus, it is likely that the MMSE is less sensitive to changes in entorhinal surface thickness in DLB, given the large sample size of DLB patients in the present study. In DLB, temporal pole surface thickness was associated with total CAMCOG and CAMCOG memory scores, most likely representing the primary memory role of this subregion (Blaizot *et al.,* 2010). The lack of association between hippocampal atrophy and common neuropsychiatric symptoms in DLB, specifically depressive symptoms and hallucinations, as well as cognitive fluctuations, reinforce arguments that these are likely to be driven by pathologies in other regions.

It is a specific strength of the study that a relatively large AD and DLB cohort was included, and that imaging data was acquired using a 3T scanner as compared with the lower field strength of previous studies (Tam *et al.,* 2005; Burton *et al.,* 2009), and that a standardised image analysis tool was used to avoid the limitations of manually delineating the hippocampus. The main limitation of the study is in the lack of concurrent amyloid biomarker imaging as a measure of AD pathology. A further limitation is the lack of longitudinal follow‐up data; longitudinal data is necessary to clarify whether hippocampal atrophy, and the atrophy of adjacent medial temporal regions, can predict the course of DLB.

Overall, despite similar levels of cognitive impairment in DLB and AD, less hippocampal atrophy was observed in DLB than AD. These results suggest that amnestic deficits in DLB may be, in part, mediated by concurrent AD pathology in the hippocampus and in the adjacent subregions of the medial temporal lobe and may have clinical implications in the stratification of DLB patients, in the future, for anti‐amyloid disease‐modifying treatments. Indirectly, these data suggest a role for non‐AD pathology or AD related pathology, focused in non‐medial temporal lobe regions in the aetiology of other cognitive domains in DLB; further work combined with amyloid imaging may help elucidate these unanswered questions.

## Conflict of interest

The authors report no conflicts of interest.

**Key points**

- It is not known how the cognitive phenotype of DLB is influenced by hippocampal atrophy or adjacent medial temporal lobe structures.
- Hippocampal atrophy and medial temporal lobe cortical thickness were associated with the severity of cognitive symptoms.
- Atrophy in these structures may partly mediate specific cognitive symptoms in DLB.

## Supporting information


*Supplementary Table S1:* Associations between total hippocampal volume and cognition in AD (*n* = 76)

*Supplementary Table S2:* Associations between medial temporal subregion cortical thickness and cognition in AD (*n* = 76)
