# Supplementary material for: The influence of hippocampal atrophy on the cognitive phenotype of dementia with Lewy bodies
Source: Int J Geriatr Psychiatry. 2017 Apr 20;32(11):1182–9. doi: 10.1002/gps.4719 (PMC5655697; doi:10.1002/gps.4719)
Supplement: Supplementary file 1 — Supplementary Table S1: Associations between total hippocampal volume and cognition in AD (n = 76) [file GPS-32-1182-s001.docx]

| *Supplementary Table 1:*  Associations between total hippocampal volume and cognition in AD (*n* = 76) | | | |
| --- | --- | --- | --- |
|  | *B* | *SE B* | *β* |
| CAMCOG total | .00 | .00 | .01 |
| CAMCOG memory | .00 | .00 | .13 |
| CAMCOG executive function | .00 | .00 | .01 |
| MMSE | .00 | .00 | .03 |

Notes: CAMCOG: Cambridge Cognitive Examination, MMSE: Mini-Mental State Examination
