# Supplementary material for: The influence of hippocampal atrophy on the cognitive phenotype of dementia with Lewy bodies
Source: Int J Geriatr Psychiatry. 2017 Apr 20;32(11):1182–9. doi: 10.1002/gps.4719 (PMC5655697; doi:10.1002/gps.4719)
Supplement: Supplementary file 2 — Supplementary Table S2: Associations between medial temporal subregion cortical thickness and cognition in AD (n = 76) [file GPS-32-1182-s002.docx]

| *Supplementary Table 2:*  Associations between medial temporal subregion cortical thickness and cognition in AD (*n* = 76) | | | |
| --- | --- | --- | --- |
|  | *B* | *SE B* | *β* |
| Total parahippocampal thickness |  |  |  |
| CAMCOG total | 3.99 | 2.94 | .17 |
| CAMCOG memory | 1.51 | 1.00 | .19 |
| CAMCOG executive function | 1.15 | 1.07 | .14 |
| MMSE | .30 | 1.01 | .04 |
| Total entorhinal thickness |  |  |  |
| CAMCOG total | .80 | 1.91 | .05 |
| CAMCOG memory | .87 | .65 | .17 |
| CAMCOG executive function | .35 | .70 | .06 |
| MMSE | .03 | .66 | .01 |
| Total temporal pole thickness |  |  |  |
| CAMCOG total | .94 | 1.63 | .07 |
| CAMCOG memory | .41 | .56 | .09 |
| CAMCOG executive function | .86 | .59 | .17 |
| MMSE | .16 | .56 | .04 |

Notes: CAMCOG: Cambridge Cognitive Examination, MMSE: Mini-Mental State Examination
